# Dextro-Quinine in Periodical Hemicrania

**Published:** 1880-04

**Authors:** C. A. Bryce


### DEXTRO-QUININE IN PERIODICAL HEMICRANIA.

BY C. A. BRYCE, M. D.

I WAS called to see a little scn of Mr. Charles Lankford of this city, several months ago, who complained of headache in the right side of his head and through the right eye. His sight was imperfect while suffering from the pain, and there was decided periodicity about the attacks, being much worse every other day; his nose would bleed very often when he was troubled with the headache. From the history of the case I regarded this as a neuralgic hemicrania of malarial origin. I accordingly prescribed quinine, iron and hyoscyamus; I found no improvement, but an increase of the head trouble with more hemorrhage from the nose. I then put him upon quinine alone; his head continued to be congested and nose would bleed frequently. I then discontinued the quinine and put him upon ergot and bromide potassium. This seemed to check the hemorrhage to some extent, but the headache and imperfect vision remained. I then discarded all remedies and put him upon 3 gr. doses of Dextro-Quinine (K. & M.) three times a day. I am pleased to report that after the second day's use of Dextro-Quinine the hemicrania was entirely relieved, nor has it since returned; the eyesight became perfect, the bleeding of the nose has occurred but once since. This boy could not take quinine without producing congestion and necessarily hemorrhage. Dextro-Quinine obviated the difficulty and cured my patient.—*Southern Clinic.*

